# An investigation of the breadth of neutralizing antibody response in cats naturally infected with feline immunodeficiency virus

**DOI:** 10.1099/vir.0.071522-0

**Published:** 2015-03

**Authors:** Paweł M. Bęczkowski, Nicola Logan, Elizabeth McMonagle, Annette Litster, Brian J. Willett, Margaret J. Hosie

**Affiliations:** 1 MRC University of Glasgow Centre for Virus Research, University of Glasgow, Glasgow, UK; 2 Small Animal Hospital, University of Glasgow, Glasgow, UK; 3 Department of Veterinary Clinical Sciences, Purdue University, West Lafayette, IN 47907, USA

## Abstract

Neutralizing antibodies (NAbs) are believed to comprise an essential component of the protective immune response induced by vaccines against feline immunodeficiency virus (FIV) and human immunodeficiency virus (HIV) infections. However, relatively little is known about the role of NAbs in controlling FIV infection and subsequent disease progression. Here, we present studies where we examined the neutralization of HIV-luciferase pseudotypes bearing homologous and heterologous FIV envelope proteins (*n* = 278) by sequential plasma samples collected at 6 month intervals from naturally infected cats (*n* = 38) over a period of 18 months. We evaluated the breadth of the NAb response against non-recombinant homologous and heterologous clade A and clade B viral variants, as well as recombinants, and assessed the results, testing for evidence of an association between the potency of the NAb response and the duration of infection, CD4^+^ T lymphocyte numbers, health status and survival times of the infected cats. Neutralization profiles varied significantly between FIV-infected cats and strong autologous neutralization, assessed using luciferase-based *in vitro* assays, did not correlate with the clinical outcome. No association was observed between strong NAb responses and either improved health status or increased survival time of infected animals, implying that other protective mechanisms were likely to be involved. Similarly, no correlation was observed between the development of autologous NAbs and the duration of infection. Furthermore, cross-neutralizing antibodies were evident in only a small proportion (13 %) of cats.

## Introduction

Neutralizing antibodies (NAbs) are elicited in response to feline immunodeficiency virus (FIV) and human immunodeficiency virus (HIV) infections, and are believed to be an essential component of the protective immune responses required for successful vaccination against lentiviruses (Kwong *et al.*, 2012). However, relatively little is known about the role of humoral immunity in controlling lentiviral infections and subsequent disease progression, particularly for FIV infection (Piantadosi *et al.*, 2009, Gray *et al.*, 2011; Hosie *et al.*, 2011), in spite of there being a vaccine available commercially that protects cats against FIV infection (Pu *et al.*, 2005).

In HIV infection, NAbs specifically target epitopes on SU and TM, including receptor- and co-receptor-binding sites (Binley *et al.*, 2008). However, their efficacy is subject to significant challenges. The viral envelope (Env) protein contains host glycans that shield neutralization epitopes on Env proteins, often rendering them inaccessible to NAbs (Myers & Lenroot, 1992). Furthermore, HIV and FIV Env proteins may display significant length polymorphisms (Kraase *et al.*, 2010; Euler & Schuitemaker, 2012) that may result in conformational changes, concealing neutralization epitopes (Hoxie, 2010).

Antibodies recognizing HIV-1 Env appear ~2 weeks after infection, but lack neutralizing activity (Tomaras & Haynes, 2009). Autologous, highly strain-specific, polyclonal NAbs appear within 3 months after infection, exert selection pressure and lead to the emergence of escape mutants (Moore *et al.*, 2008; Li *et al.*, 2009). It has been documented in HIV-1 infection that autologous NAbs have little or no protective effect on disease progression (Bunnik *et al.*, 2008; Mahalanabis *et al.*, 2009; van Gils *et al.*, 2010; Gray *et al.*, 2011), largely as a result of the rapid emergence of escape mutants (Bunnik *et al.*, 2010; van Gils *et al.*, 2010). The emergence of neutralization escape mutants with altered glycosylation patterns has been demonstrated both in HIV (Burton *et al.*, 2005; van Gils *et al.*, 2010) and FIV (Samman *et al.*, 2010) infections. Neutralization escape, accompanied by the subsequent evolution of the antibody response, occurs during the course of infection in response to the evolving viral Env, until the eventual exhaustion of the immune system (Euler & Schuitemaker, 2012). This explains why NAbs from a specific time point can neutralize viruses isolated from earlier time points, but fail to neutralize contemporaneous viral variants (Mascola & Montefiori, 2010; Overbaugh & Morris, 2012).

It has been suggested that NAbs appear too late following infection with HIV-1 to be effective in controlling disease progression (Richman *et al.*, 2003; Moore *et al.*, 2009; Rong *et al.*, 2009). However, NAbs have been shown to have a potential role in controlling simian immunodeficiency virus (SIV)–HIV (SHIV) infection of macaques depleted of cytotoxic T lymphocytes (Rasmussen *et al.*, 2002). Furthermore, pre-exposure passive transfer of broadly neutralizing mAbs conferred protection against SIV and SHIV-1 in the rhesus macaque model (Veazey *et al.*, 2003; Ferrantelli *et al.*, 2004; Hessell *et al.*, 2009), providing evidence that NAbs do indeed play a protective role and are likely an essential component of a protective vaccine response (Hoxie, 2010).

HIV infection, in the majority of patients, leads to the robust production of antibodies that often possess the ability to neutralize autologous but not heterologous viral variants (Zolla-Pazner *et al.*, 2004). Broadly cross-reactive NAbs (Cr-NAbs) are relatively rare; elicited in some individuals, Cr-NAbs neutralize not only autologous viral variants, but also neutralize other viral subtypes (Walker *et al.*, 2011). Several broadly neutralizing mAbs have been isolated (including b12, 2G12, 2F5 and 4E10), their binding epitopes have been characterized (Muster *et al.*, 1993; Burton *et al.*, 1994; Gorny *et al.*, 1994; Trkola *et al.*, 1996; Zwick *et al.*, 2001) and protective roles in animal models have been demonstrated (Mascola *et al.*, 1999; Mascola *et al.*, 2000; Binley *et al.*, 2004). Recent evidence suggests that Cr-NAbs are more common than previously estimated, arising in approximately one-third of HIV-1-infected individuals (Stamatatos *et al.*, 2009; Bonsignori *et al.*, 2011; Medina-Ramírez *et al.*, 2011; Mikell *et al.*, 2011; Walker *et al.*, 2011; Euler & Schuitemaker, 2012). However, neutralization breadth does not develop until ~3 years post-infection (Gray *et al.*, 2011; Mikell *et al.*, 2011). It remains unknown why, and by which mechanism, such antibodies develop in some individuals or why the broadly neutralizing response is significantly delayed in response to infection (Gray *et al.*, 2011). Furthermore, it is unclear whether neutralization cross-reactivity can be attributed to a single, highly potent antibody or a combination of antibodies acting in synergy (Scheid *et al.*, 2009).

The strength and breadth of the NAb response was greater in HIV progressors compared with aviraemic or long-term non-progressors (Doria-Rose *et al.*, 2009). Studies of elite controllers revealed that individuals who controlled viral replication, such that their viraemia was below detectable levels, had the lowest levels of NAbs (Lambotte *et al.*, 2009; Pereyra *et al.*, 2009). Consistent with this observation, the breadth of Cr-NAbs was positively correlated with higher plasma viral loads, lower CD4^+^ T lymphocyte counts and disease progression (Piantadosi *et al.*, 2009; Sather *et al.*, 2009; van Gils *et al.*, 2009; Euler *et al.*, 2010). These results suggested that the development of Cr-NAbs is influenced by strong antigenic stimulation (Gray *et al.*, 2011). However, individuals who did not develop Cr-NAbs might have failed to do so as a result of insufficient antigenic stimulation and possibly non-specific hypergammaglobulinaemia (Euler & Schuitemaker, 2012). Despite the breadth and potency of Cr-NAbs *in vitro*, such antibodies do not appear to influence HIV-1 disease progression; rather, their incremental development is associated with increased viral loads and declining numbers of CD4^+^ T lymphocytes (Piantadosi *et al.*, 2009; van Gils *et al.*, 2009; Euler *et al.*, 2010; Gray *et al.*, 2011).

Little is known about the role of NAbs in controlling natural FIV infection and subsequent disease progression (Hosie *et al.*, 2011), although NAbs appear to be involved in vaccine-induced protective immunity (Hosie & Flynn, 1996; Pu *et al.*, 2001). What is the relationship between the duration of infection, health status, survival time and the NAb response in FIV-infected cats? Can a strong NAb response delay disease progression? Is there evidence for broadly Cr-NAbs in plasma samples from naturally infected cats? Although the gold standard neutralization assay utilizes primary PBMCs as target cells, as well as uncloned primary field isolates, the reproducibility of such systems is limited by the variability in susceptibility of PBMCs to infection, as reviewed previously (Hosie *et al.*, 2011). Therefore, in this study we utilized a pseudotype-based neutralization assay, similar to the assay systems that have been shown to be robust and highly reproducible for measuring HIV neutralization, to measure NAb responses in cats naturally infected with FIV in order to investigate the role of neutralization.

## Results

### FIV-infected cats display variable neutralization patterns

Plasma samples from 38 cats displayed variable autologous and heterologous neutralization patterns, ranging from strong, through moderate to no neutralization (Table S1, available in the online Supplementary Material). Plasma samples from 16 cats (16/38, 42.1 %) strongly neutralized pseudotypes bearing autologous Env variants. This pattern was observed in eight (8/16, 50 %) of the cats that remained alive for the duration of the study and eight (8/16, 50 %) of the cats that died during the study. Six cats (6/38, 15.8 %) displayed moderate neutralization of pseudotypes bearing autologous Env variants; all but one of these cats remained alive during the observation period. Plasma samples from 16 cats (16/38, 42.1 %) failed to neutralize pseudotypes bearing autologous Env variants; nine of these cats (9/16, 56 %) remained alive, whereas seven (7/16, 44 %) of the cats with no detectable NAbs died during the study period. Only five cats (5/38, 13 %) demonstrated strong heterologous neutralization of at least one pseudotype; all of those cats remained alive during the study.

### Autologous NAbs and duration of infection

**Fig. 1.**
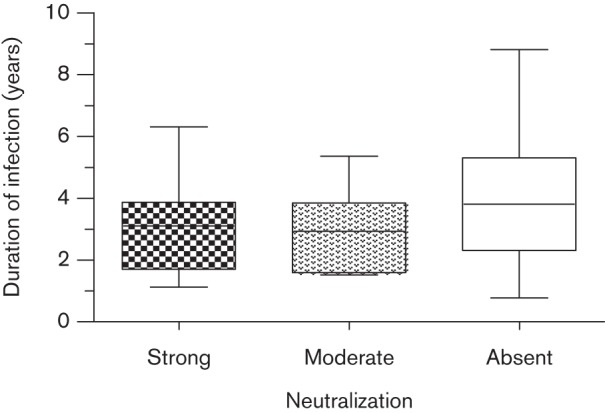
Relationship between the duration of infection and the development of autologous NAbs. Duration of infection for cats with strong (*n* = 16), moderate (*n* = 6) and absent (*n* = 16) autologous neutralization responses is shown from left to right (median 3.1, 2.9 and 3.8 years, respectively).

We investigated whether the development of autologous neutralization was positively correlated with the age of the cats and the duration of infection. Fig. 1 illustrates the relationship between the potency of autologous NAb response and the duration of infection for cats from the entire study group. The median duration of infection for cats with strong, moderate and absent autologous NAb responses was 3.1 (1.1–6.3), 2.9 (1.5–5.4) and 3.8 (range 0.8–8.8) years, respectively. No statistically significant differences between the groups were observed.

### NAb responses in cats infected with recombinant and non-recombinant viruses

**Fig. 2.**
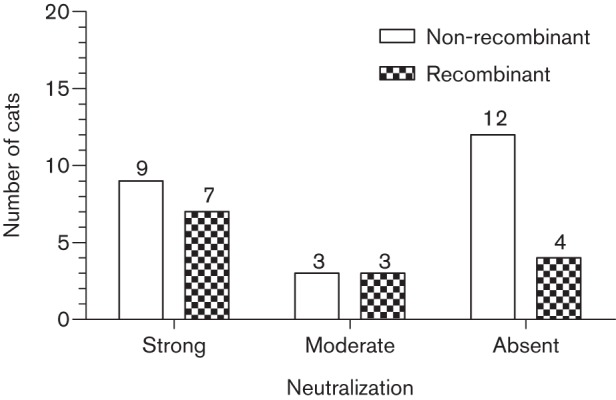
Autologous neutralization responses in cats infected with non-recombinant (*n* = 24) and recombinant (*n* = 14) *env* viruses. Entire *env* sequences from the study group (*n* = 355), together with reference full-length *env* sequences obtained from GenBank (*n* = 19), were subjected to rigorous fivefold recombination testing as described previously (Bęczkowski *et al.*, 2014).

We hypothesized that cats infected with recombinant *env* viruses (*n* = 14) would have more potent autologous and heterologous NAb responses than cats infected with non-recombinant *env* viruses (*n* = 24). However, no statistically significant differences were observed between the strength of autologous neutralization in cats infected with recombinant compared with non-recombinant viruses (Fig. 2).

Next, we asked whether cross-reactive NAb responses were more common in cats infected with recombinant *env* viruses compared with cats infected with non-recombinant *env* viruses. There was no statistically significant difference between the two groups; of five cats that demonstrated heterologous neutralization (against at least one pseudotype), three were infected with non-recombinant viruses, whilst two were infected with recombinant viruses.

### NAb response and health status of infected animals

**Fig. 3.**
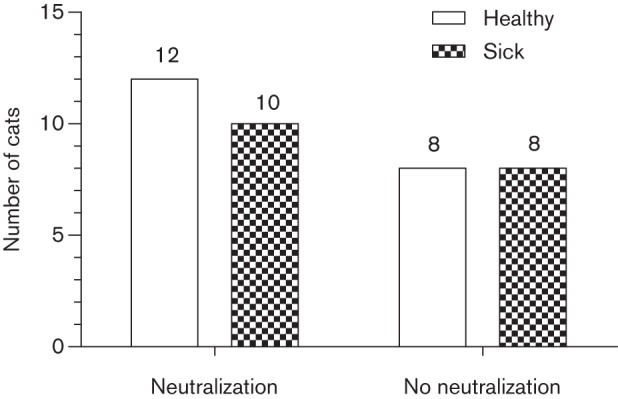
NAb responses according to health status. Within the group of cats expressing strong and moderate autologous NAb responses (*n* = 22), there were 12 healthy and 10 sick cats. Within the group with no autologous neutralization (*n* = 16), there were eight healthy and eight sick cats.

We examined the data for an association between the presence of autologous NAbs and the health status of infected cats (Fig. 3). Health status was assessed by a board-certified feline medicine specialist, but was nevertheless subjective, and so we also examined the data to test for an association between autologous NAbs and declining CD4^+^ T lymphocyte numbers. As demonstrated in Table 1, all but two of the cats within the Memphis cohort (*n* = 24) demonstrated a progressive decline in CD4^+^ T lymphocytes. The median ΔCD4^+^ over the 18 month observation period was –340 cells µl^−1^ (ranging from −1120 to +30 cells µl^−1^). In contrast, within the Chicago cohort (*n* = 14), seven cats displayed progressive declines in CD4^+^ T lymphocyte numbers, five displayed increased numbers, whilst two cats maintained their CD4^+^ T lymphocyte numbers over a period of 12 months (Table 1). The median ΔCD4^+^ was calculated as −15 cells µl^−1^ (ranging from –760 to +240 cells µl^−1^).

**Table 1.** CD4^+^ T lymphocyte counts for each time point (A, B, C and D) unless a cat was deceased (X) or a sample was not available (na) ΔCD4^+^ in the final column represents the difference between the first (A) and the last available sampling. All but two cats from the Memphis cohort (M) displayed a progressive decline in CD4^+^ T lymphocyte numbers over the 18-month observation period. Five cats from the Chicago cohort (P) (5/14) displayed increasing CD4^+^ T lymphocyte numbers over the 12 month observation period.

Cat	CD4^+^ count (×10^3^ cells µl^−1^)				ΔCD4^+^ (×10^3^ cells µl^−1^)	Cat	CD4^+^ count (×10^3^ cells µl^−1^)				ΔCD4^+^ (×10^3^ cells µl^−1^)
	A	B	C	D			A	B	C	D
M2	1.74	0.36	0.76	0.62	−1.12	M5	0.56	0.15	0.16	X	−0.40
M29	1.48	0.62	0.93	0.62	−0.86	M50	1.38	1.32	X	X	−0.07
M1	0.87	0.36	0.34	0.26	−0.61	M33	0.20	X	X	X	na
M15	0.87	1.03	1.09	0.47	−0.40	M3	0.33	X	X	X	na
M8	0.55	0.21	0.2	0.15	−0.40	M44	1.24	X	X	X	na
M49	0.41	0.31	0.27	0.04	−0.38	P4	0.09	0.34	0.33	na	0.24
M28	1.23	0.89	1.77	0.90	−0.34	P14	0.97	1.16	1.09	na	0.12
M14	0.45	0.29	0.15	0.14	−0.30	P8	0.50	0.86	0.60	na	0.10
M25	0.36	0.39	0.68	0.09	−0.27	P6	0.48	0.35	0.57	na	0.09
M20	1.50	0.81	na	1.25	−0.25	P7	0.40	0.26	0.40	na	0
M47	0.29	0.14	0.14	0.10	−0.19	P11	0.45	0.35	0.45	na	0
M32	0.38	0.32	0.35	0.21	−0.17	P13	0.46	0.28	0.27	na	−0.18
M30	0.13	0.15	0.26	0.10	−0.04	P17	0.49	0.55	0.28	na	−0.21
M46	0.15	0.19	0.18	0.18	0.03	P9	0.63	0.35	0.18	na	−0.45
M11	0.98	0.14	0.48	X	−0.50	P5	0.79	0.72	0.30	na	−0.49
M16	0.35	0.37	0.36	X	0.01	P2	0.40	0.36	X	na	−0.03
M26	0.54	0.14	0.13	X	−0.41	P21	na	0.93	0.57	na	−0.36
M31	0.80	0.88	0.26	X	−0.55	P22	na	1.55	0.79	na	−0.76
M41	0.34	0.35	0.12	X	−0.22	P18	na	0.73	0.75	na	0.02

**Fig. 4.**
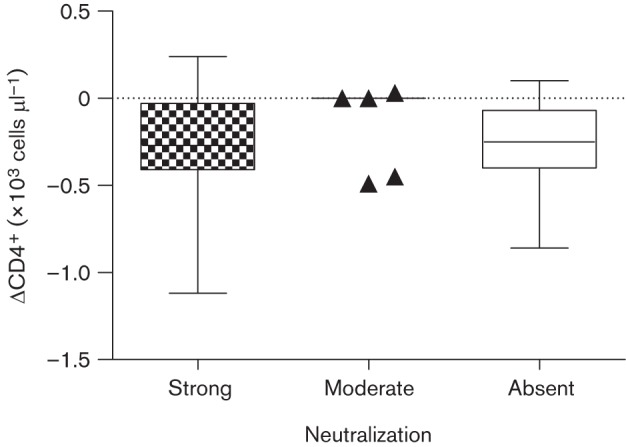
Changes in CD4^+^ lymphocyte count (×10^3^ cells µl^−1^) over the course of infection in cats with strong (*n* = 15; median −0.27, range −1.12 to +0.24), moderate (*n* = 5; median 0.0, range −0.49 to +0.03) or absent (*n* = 15; median −0.25, range −0.86 to +0.10) NAb responses. ΔCD4^+^ values were not available for three cats (Table 1).

We then asked whether a strong autologous NAb response might protect cats against a progressive decline in CD4^+^ T lymphocytes. We compared three groups of cats: those with absent (*n* = 15), moderate (*n* = 5) or strong (*n* = 15) NAb responses for which ΔCD4^+^ values were available (*n* = 35). ΔCD4^+^ values were not available for three cats (3/38; M3, M33 and M44) as these cats died prior to the second blood sampling (Table 1). As demonstrated in Fig. 4, there was no evidence that cats with strong NAb responses were less likely to display progressively declining CD4^+^ T lymphocyte numbers (median ΔCD4 = –270 cells µl^−1^); a similar range of ΔCD4^+^ values was observed within the group of cats which failed to mount autologous NAb responses (median ΔCD4 = –250 cells µl^−1^) and there were no statistically significant differences between the two groups. Three animals with moderate levels of NAbs maintained their CD4^+^ T lymphocyte numbers, whilst two animals showed declining CD4^+^ T lymphocyte numbers.

### NAb response and survival time of infected animals

**Fig. 5.**
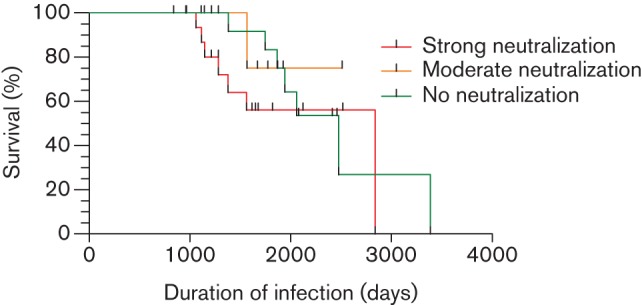
Kaplan–Meyer survival curves for cats with strong (*n* = 16) (red), moderate (*n* = 6) (orange) or weak/absent (*n* = 16) (green) autologous neutralization.

We examined the relationship between the autologous neutralization responses and survival times of infected cats since the estimated time of infection. Kaplan–Meyer survival curves were constructed for three groups of cats expressing strong (*n* = 16), moderate (*n* = 6) or no (*n* = 16) autologous neutralization (Fig. 5).

The estimated median survival time for cats with strong autologous NAb responses was 2840 (range 1061–2840) days and for the group with no NAbs was 2476 (range 1384–3387) days. There was insufficient data within the moderate neutralization group to estimate median survival. A comparison of the Kaplan–Meyer curves [log-rank (Mantel–Cox) test] revealed that survival times were not significantly different amongst the three groups (*P* = 0.48). Further testing (log-rank test for trend) revealed no significant trend between the three survival curves (*P* = 0.36).

### NAbs in the terminal stages of disease

Finally, we asked whether the NAb response was preserved in the terminal stages of disease, when the immune system was weakened. There were 13 cats in the study group with CD4^+^ T lymphocyte counts <200 cells µl^−1^ recorded at the final sampling (Table 1), indicative of terminal infection (by analogy to HIV infection). Plasma samples from six (6/13; 46 %) of these cats did not neutralize pseudotypes bearing homologous Env proteins, whilst plasma samples from seven (7/13; 54 %) of the cats neutralized pseudotypes bearing autologous Env proteins, in spite of the cats being assumed to be in the terminal stage of disease.

## Discussion

We demonstrated that cats naturally infected with FIV have variable NAb responses against pseudotypes bearing autologous and heterologous Env proteins. When neutralization assays were conducted against pseudotypes bearing Env proteins from autologous and heterologous viruses, no correlation was evident between either the health status or the survival time following infection and the strength of the NAb response. Similar neutralization profiles were observed for plasma samples tested from healthy and unhealthy cats as well as cats that survived or died during the study period. Such findings argue against a role for NAbs in controlling disease progression. Furthermore, our results demonstrate that FIV-infected cats, regardless of the strength of the NAb response induced, showed progressive declines in CD4^+^ T lymphocyte numbers; NAb responses, even when potent, did not appear to protect against the loss of CD4^+^ T lymphocytes. A similar trend has been reported for HIV-1 infection, where the presence of autologous, Cr-NAbs was not associated with a prolonged AIDS-free, asymptomatic period (Schmitz *et al.*, 2003; Piantadosi *et al.*, 2009; van Gils *et al.*, 2010; Euler *et al.*, 2010). Several studies have suggested that neutralization breadth and potency depend on the duration of infection (Moog *et al.*, 1997; Deeks *et al.*, 2006; Sather *et al.*, 2009). However, the results presented here do not support this proposal; rather, no association was observed between the duration of infection and the potency of autologous neutralization.

Almost half of the cats examined displayed strong autologous NAb responses; 24 % of the cats showed a steady increase in neutralization potency during the course of infection. A similar increase in the strength of autologous NAb response has been reported for HIV-1 infection (Arendrup *et al.*, 1992; Geffin *et al.*, 2003; Richman *et al.*, 2003). Although NAbs failed to protect against contemporaneous viruses, it was suggested that such antibodies might exert selection pressure on the emergence of viral variants of lower fitness, e.g. with decreased replicative capacity, and thus might indirectly delay HIV-1-associated disease progression (Friedrich *et al.*, 2004; Leslie *et al.*, 2004). However, in this study we found no evidence of any correlation between the strength of NAb response and survival time in cats naturally infected with FIV.

The remaining half of the cats that were examined did not develop NAbs. As a high level of antigenic stimulation is crucial for the development of broad and potent NAb responses (Rodriguez *et al.*, 2007; Doria-Rose *et al.*, 2009; Sather *et al.*, 2009), it is possible that the immune systems of those cats that did not neutralize pseudotypes bearing autologous Env proteins had not been exposed to sufficient antigenic stimulation following infection to induce NAbs. This is most likely the case with cat M1, which acquired the virus vertically (Bęczkowski, 2013) and failed to mount a NAb response. Given that a high viral load and high viral diversity following infection influence the development of potent and broad NAbs (Piantadosi *et al.*, 2009; Sather *et al.*, 2009; Euler *et al.*, 2010; Gray *et al.*, 2011), it is tempting to speculate that those cats in our study group which failed to develop NAb responses may have had relatively low viral load set points compared to the cats which developed NAbs. Unfortunately, viral load set point data following the postulated transmission events were not available to test this hypothesis.

Furthermore, the non-specific CD4^+^ T lymphocyte-dependent polyclonal hypergammaglobulinaemia that arises as an initial response to FIV infection (Recher *et al.*, 2004; Lang *et al.*, 2007) might also contribute to the lack of effective neutralization observed in this group. It is possible that high numbers of CD4^+^ T lymphocytes at the time of virus acquisition might be responsible for a non-specific, overwhelming hypergammaglobulinaemia and subsequent failure of NAb responses to develop (Euler *et al.*, 2010; Gray *et al.*, 2011). This scenario could also explain why only a small fraction of cats in our study demonstrated cross-neutralization of the heterologous GL-8 and B2542 pseudotypes, regardless of whether they were infected with recombinant or non-recombinant viruses.

A study examining the breadth of neutralization in a similar number of HIV-1-infected individuals (*n* = 40) revealed that 17.5 % of patients developed broadly NAbs (Gray *et al.*, 2011). Other studies reported higher numbers (up to 30 %) of individuals with broadly NAbs (Doria-Rose *et al.*, 2009; Gray *et al.*, 2009; Piantadosi *et al.*, 2009; Sather *et al.*, 2009; Euler *et al.*, 2010). In contrast, only 13 % of plasma samples from our study group displayed cross-reactivity. This may suggest that Cr-NAbs are rarer in FIV-infected cats than in HIV-1-infected individuals. However, a limitation of this study was that plasma samples were tested for neutralization against only two reference pseudotypes bearing heterologous FIV Env proteins; it is possible that testing a greater number of pseudotypes bearing Env proteins from more strains of FIV might have revealed a higher prevalence of Cr-NAbs. In studies of HIV, it has been suggested that neutralization breadth develops slowly over a period of 2–4 years post-seroconversion (Gray *et al.*, 2011; Mikell *et al.*, 2011). Given the duration of infection in our study group, it would be predicted, by analogy, that more cats would have developed Cr-NAbs, but this scenario was not supported by the data presented here.

Finally, differences in neutralization profiles might have been related to different kinetics of viral replication between the various strains of FIV infecting the cats. It is possible that more virulent, and more replication competent, viral strains are more likely to induce effective humoral responses compared with isolates of lower replicative capacity.

The results presented here demonstrate that humoral immunity was preserved in cats that subsequently developed AIDS, consistent with the observation that the rate of viral evolution slows during the terminal stage of disease (Bęczkowski, 2013). Thus, autologous antibodies elicited during the earlier stages of infection remain capable of neutralization, owing to the relatively high genetic stability of the virus terminally (Bęczkowski, 2013). However, such NAbs, although capable of neutralization *in vitro* and despite being preserved in terminal disease, failed to prevent disease progression.

Pseudotypes bearing 1–18 Env variants from each time point were used to assess sensitivity to neutralization by autologous plasma. It is difficult to assess how representative the cloned Env proteins were compared to the pool of Env variants within the cats. Nevertheless, the alternative approach of testing a single ‘representative’ clonal Env variant would have led to an underestimation of viral diversity within the host. The sensitivities to neutralization amongst pseudotypes bearing Env variants isolated from each cat tended to be similar; these data suggest that, where changes were observed, these were likely a true indication of the range of neutralization sensitivity and resistance amongst the pool of variants in individual cats.

Any assay system used to assess NAb responses *in vitro* will be limited in its representation of *in vivo* neutralization. Nevertheless, the indicator cell line transduced with CD134 that was selected for use in this study displayed a pattern of susceptibility to infection consistent with that of the MYA-1 cell line, an IL-2-dependent, CD4^+^ feline T cell line expressing CD134 and CXCR4 that has been utilized previously in neutralization assays (Hosie *et al.*, 2011). This suggests that the pattern of CD134 and CXCR4 expression on the indicator cell line recapitulates the cell surface phenotype of the IL-2-dependent primary T cell line used formerly in neutralization assays.

We demonstrated that humoral immunity did not significantly alter the clinical course of natural FIV infection and, although this study was limited by the size of the cohort examined, and thus the statistical power obtained, it provides the basis for future studies. One explanation for this apparent lack of correlation is that any factor promoting a strong NAb response may negatively influence other immune responses, e.g. leading to the exhaustion of polyfunctional CD4^+^ and CD8^+^ T lymphocytes (Harari *et al.*, 2004; Betts *et al.*, 2006; Streeck *et al.*, 2008). In light of recent evidence from studies with HIV-1, it is plausible that cell-mediated immunity, as well as host genetic factors, are more likely to influence the clinical course of lentiviral infection than NAbs (Huang *et al.*, 2012; Nomura & Matano, 2012). It will be intriguing to evaluate the performance of FIV vaccination in the field by measuring the development of NAbs in vaccinated cats, in order to determine whether NAbs are protective in the face of natural challenge.

## Methods

### 

#### Cats and plasma samples.

Forty-four cats from Memphis, TN, USA (*n* = 27) and Chicago, IL, USA (*n* = 17) were enrolled in the study on the basis of a history of FIV infection, regardless of breed, sex, age and health status (Bęczkowski, 2013). Twenty-seven of the FIV-positive cats enrolled were housed together in a large multi-cat household in Memphis. The remaining 17 FIV-positive cats had been previously adopted from a large metropolitan adoption-guarantee shelter (PAWS Chicago) and lived in single-cat households in Chicago, except for seven cats: two cats (P7 and P4) cohabited in a two-cat household, one cat (P13) lived in a two-cat household with a FIV-negative cat, one cat (P9) was housed at PAWS Chicago for the first 11 weeks of the study and then was adopted into a house with an FIV-positive cat not enrolled in the study, and three cats (P2, P15 and P21) were housed at PAWS Chicago in a room containing up to three FIV-positive cats before they were each adopted into single-cat households at 2, 14 and 58 weeks after enrolment, respectively. The FIV status of each cat was confirmed by virus isolation (Hosie *et al.*, 2009). All cats were feline leukemia virus antigen-negative at enrolment. Four blood samples (denoted A, B, C and D, related to each collection time point) were obtained from each cat at 6 month intervals over an 18 month period, unless the cat had died during the interim period. During the study, one of 17 (5.9 %) cats from the Chicago cohort and 17 of 27 (63 %) FIV-positive cats from the Memphis cohort died. Analysis of CD4^+^ and CD8^+^ T lymphocyte subsets (Table 1) and post-mortem findings (Bęczkowski, 2013) suggested that, in the majority of cases, FIV infection played a role in the observed morbidity and mortality. Detailed recording of signallement, clinical history, physical examination data and body weight, and flow cytometry analysis of CD4^+^ and CD8^+^ lymphocyte subsets were performed at the time of each sampling (Bęczkowski, 2013), and are summarized in Table S2.

The study and its aims were reviewed and approved by the University of Glasgow Ethics Committee and the Purdue Animal Care and Use Committee. Cat owners provided written informed consent for their participation in the study.

#### Amplification and cloning of WT FIV *env* genes.

Full-length FIV *env* genes (~2500 bp) were amplified from whole-blood samples using a two-step nested PCR protocol. First-round PCRs were performed using Phusion Blood Direct II Polymerase (Thermo Fisher Scientific) followed by direct nucleic acid sequence determination. The nucleic acid sequence of the first-round PCR product informed primer design for the second-round PCR, which was performed using Roche High Fidelity Master (Roche); strain-specific primers for second-round PCR incorporated restriction sites to facilitate subcloning into the expression vector for pseudotyping (Table S3). In addition, reference *env* genes from clade A (GL-8) and clade B (B2542) were cloned into the eukaryotic expression vector VR1012 (Hartikka *et al.*, 1996) and transformed into *Escherichia coli* MAX Efficiency DH5α Competent Cells (Invitrogen). Next, VR1012 plasmids expressing FIV *env* genes were transiently co-transfected with the HIV pNL4-3-Luc-E^−^R^−^luc plasmid (an *env*-deleted HIV provirus containing the luciferase reporter gene) (Connor *et al.*, 1995) into HEK 293T cells (Graham *et al.*, 1977), using Superfect Transfection Reagent (Invitrogen). Following 72 h incubation in six-well culture clusters (Corning), culture fluids containing pseudoviruses were harvested, centrifuged at 1000 r.p.m. (~200 ***g***) for 5 min, passed through 0.45 µm filters and stored at −80 °C until required.

In this way, pseudotypes (*n* = 278) were prepared, bearing naturally occurring (*n* = 276) and reference (*n* = 2) FIV Env proteins on an HIV backbone; the single-round, replication-competent pseudoviruses were used to assess the neutralization properties of test plasma samples. Pseudotypes were prepared bearing Env proteins from 38 cats (38/44, 86.4 %); it was not possible to produce viable pseudoviruses bearing Env proteins from five cats (5/44, 11.4 %; largely because of premature stop codons occurring in the *env* sequences) and plasma samples from one cat were not available for testing.

#### Neutralization assay.

Plasma samples from 38 cats were tested for NAbs using HIV(FIV)luc pseudotypes. Plasma samples were heat inactivated at 56 °C for 30 min in order to inactivate complement and diluted 10-fold from a starting dilution of 1 : 10 in complete RPMI 1640 medium (Invitrogen). For 1 h at 37 °C, 25 µl of each plasma dilution (1 : 10, 1 : 100 and 1 : 1000) were incubated in triplicate with 25 µl HIV(FIV)luc pseudotype [luciferase activity on CLL-CD134 cells (Willett *et al.*, 2006) of ~5×10^7^ c.p.m.] before 5×10^4^ CLL-CD134 cells were added in 50 µl and cultured in CulturPlate-96 assay plates (Perkin Elmer) for 72 h. Next, luciferase activity was quantified following the addition of 100 µl Steadylite HTS (Perkin Elmer) substrate and single-photon counting was conducted using a MicroBeta luminometer (Perkin Elmer).

The neutralization activity of the tested plasma samples is presented as ‘fold neutralization’. Fold neutralization was calculated by dividing the mean luciferase counts of control wells containing no plasma (NP luc) by the mean luciferase counts for wells containing 1 : 10 plasma dilutions (P luc). Fold neutralization may be compared with the percentage neutralization calculated according to:

$$\text{Neutralization}\;\left(\%\right)\;=\frac{\left(\text{NP}\;\text{luc}\;-\;\text{P}\;\text{luc}\right)}{\text{NP}\;\text{luc}}\;\times \;100$$

Plasma samples were classified as not neutralizing, or weakly, moderately or strongly neutralizing according to the empirical cut-off values shown in Table S4.

#### Graphs and statistical analyses.

Graphs and statistical analyses were performed in Prism version 5.00 (GraphPad Software). Descriptive data were shown as medians and interquartile range (fifth and 95th quartile). Binary data were analysed using Fisher’s exact test. Kaplan–Meier curves were compared using the Mantel–Cox ‘log-rank’ test and tested with the log-rank test for trends. Significance was set at *P*<0.05. For clarity, values for fold neutralization at 1 : 10 plasma dilutions are shown in Table S1.

## References

[r1] Arendrup M. Nielsen C. Hansen J. E. Pedersen C. Mathiesen L. Nielsen J. O. **(**1992**).** Autologous HIV-1 neutralizing antibodies: emergence of neutralization-resistant escape virus and subsequent development of escape virus neutralizing antibodies. J Acquir Immune Defic Syndr 5, 303–307. 10.1097/00126334-199203000-00012 1740756

[r2] **Bęczkowski, P. M. (2013).** *Virus evolution in the progression of natural feline immunodeficiency virus infection*. PhD thesis, University of Glasgow, UK.

[r3] Bęczkowski P. M. Hughes J. Biek R. Litster A. Willett B. J. Hosie M. J. **(**2014**).** Feline immunodeficiency virus (FIV) *env* recombinants are common in natural infections. Retrovirology 11, 80 10.1186/s12977-014-0080-1 PMC4180853 25699660

[r4] Betts M. R. Nason M. C. West S. M. De Rosa S. C. Migueles S. A. Abraham J. Lederman M. M. Benito J. M. Goepfert P. A. **& other authors (**2006**).** HIV nonprogressors preferentially maintain highly functional HIV-specific CD8^+^ T cells. Blood 107, 4781–4789. 10.1182/blood-2005-12-4818 16467198 PMC1895811

[r5] Binley J. M. Wrin T. Korber B. Zwick M. B. Wang M. Chappey C. Stiegler G. Kunert R. Zolla-Pazner S. **& other authors (**2004**).** Comprehensive cross-clade neutralization analysis of a panel of anti-human immunodeficiency virus type 1 monoclonal antibodies. J Virol 78, 13232–13252. 10.1128/JVI.78.23.13232-13252.2004 15542675 PMC524984

[r6] Binley J. M. Lybarger E. A. Crooks E. T. Seaman M. S. Gray E. Davis K. L. Decker J. M. Wycuff D. Harris L. **& other authors (**2008**).** Profiling the specificity of neutralizing antibodies in a large panel of plasmas from patients chronically infected with human immunodeficiency virus type 1 subtypes B and C. J Virol 82, 11651–11668. 10.1128/JVI.01762-08 18815292 PMC2583680

[r7] Bonsignori M. Hwang K. K. Chen X. Tsao C. Y. Morris L. Gray E. Marshall D. J. Crump J. A. Kapiga S. H. **& other authors (**2011**).** Analysis of a clonal lineage of HIV-1 envelope V2/V3 conformational epitope-specific broadly neutralizing antibodies and their inferred unmutated common ancestors. J Virol 85, 9998–10009. 10.1128/JVI.05045-11 21795340 PMC3196428

[r8] Bunnik E. M. Pisas L. van Nuenen A. C. Schuitemaker H. **(**2008**).** Autologous neutralizing humoral immunity and evolution of the viral envelope in the course of subtype B human immunodeficiency virus type 1 infection. J Virol 82, 7932–7941. 10.1128/JVI.00757-08 18524815 PMC2519599

[r9] Bunnik E. M. Lobbrecht M. S. van Nuenen A. C. Schuitemaker H. **(**2010**).** Escape from autologous humoral immunity of HIV-1 is not associated with a decrease in replicative capacity. Virology 397, 224–230. 10.1016/j.virol.2009.11.009 19945135

[r10] Burton D. R. Pyati J. Koduri R. Sharp S. J. Thornton G. B. Parren P. W. Sawyer L. S. Hendry R. M. Dunlop N. **& other authors (**1994**).** Efficient neutralization of primary isolates of HIV-1 by a recombinant human monoclonal antibody. Science 266, 1024–1027. 10.1126/science.7973652 7973652

[r11] Burton D. R. Stanfield R. L. Wilson I. A. **(**2005**).** Antibody vs. HIV in a clash of evolutionary titans. Proc Natl Acad Sci U S A 102, 14943–14948. 10.1073/pnas.0505126102 16219699 PMC1257708

[r12] Connor R. I. Chen B. K. Choe S. Landau N. R. **(**1995**).** Vpr is required for efficient replication of human immunodeficiency virus type-1 in mononuclear phagocytes. Virology 206, 935–944. 10.1006/viro.1995.1016 7531918

[r13] Deeks S. G. Schweighardt B. Wrin T. Galovich J. Hoh R. Sinclair E. Hunt P. McCune J. M. Martin J. N. **& other authors (**2006**).** Neutralizing antibody responses against autologous and heterologous viruses in acute versus chronic human immunodeficiency virus (HIV) infection: evidence for a constraint on the ability of HIV to completely evade neutralizing antibody responses. J Virol 80, 6155–6164. 10.1128/JVI.00093-06 16731954 PMC1472617

[r14] Doria-Rose N. A. Klein R. M. Manion M. M. O’Dell S. Phogat A. Chakrabarti B. Hallahan C. W. Migueles S. A. Wrammert J. **& other authors (**2009**).** Frequency and phenotype of human immunodeficiency virus envelope-specific B cells from patients with broadly cross-neutralizing antibodies. J Virol 83, 188–199. 10.1128/JVI.01583-08 18922865 PMC2612342

[r15] Euler Z. Schuitemaker H. **(**2012**).** Cross-reactive broadly neutralizing antibodies: timing is everything. Front Immunol 3, 215. 10.3389/fimmu.2012.00215 22833745 PMC3400945

[r16] Euler Z. van Gils M. J. Bunnik E. M. Phung P. Schweighardt B. Wrin T. Schuitemaker H. **(**2010**).** Cross-reactive neutralizing humoral immunity does not protect from HIV type 1 disease progression. J Infect Dis 201, 1045–1053. 10.1086/651144 20170371

[r17] Ferrantelli F. Rasmussen R. A. Buckley K. A. Li P. L. Wang T. Montefiori D. C. Katinger H. Stiegler G. Anderson D. C. **& other authors (**2004**).** Complete protection of neonatal rhesus macaques against oral exposure to pathogenic simian-human immunodeficiency virus by human anti-HIV monoclonal antibodies. J Infect Dis 189, 2167–2173. 10.1086/420833 15181562

[r18] Friedrich T. C. Dodds E. J. Yant L. J. Vojnov L. Rudersdorf R. Cullen C. Evans D. T. Desrosiers R. C. Mothé B. R. **& other authors (**2004**).** Reversion of CTL escape-variant immunodeficiency viruses *in vivo*. Nat Med 10, 275–281. 10.1038/nm998 14966520

[r19] Geffin R. Hutto C. Andrew C. Scott G. B. **(**2003**).** A longitudinal assessment of autologous neutralizing antibodies in children perinatally infected with human immunodeficiency virus type 1. Virology 310, 207–215. 10.1016/S0042-6822(03)00137-5 12781708

[r20] Gorny M. K. Moore J. P. Conley A. J. Karwowska S. Sodroski J. Williams C. Burda S. Boots L. J. Zolla-Pazner S. **(**1994**).** Human anti-V2 monoclonal antibody that neutralizes primary but not laboratory isolates of human immunodeficiency virus type 1. J Virol 68, 8312–8320. 7525987 10.1128/jvi.68.12.8312-8320.1994 PMC237300

[r21] Graham F. L. Smiley J. Russell W. C. Nairn R. **(**1977**).** Characteristics of a human cell line transformed by DNA from human adenovirus type 5. J Gen Virol 36, 59–72. 10.1099/0022-1317-36-1-59 886304

[r22] Gray E. S. Taylor N. Wycuff D. Moore P. L. Tomaras G. D. Wibmer C. K. Puren A. DeCamp A. Gilbert P. B. **& other authors (**2009**).** Antibody specificities associated with neutralization breadth in plasma from human immunodeficiency virus type 1 subtype C-infected blood donors. J Virol 83, 8925–8937. 10.1128/JVI.00758-09 19553335 PMC2738176

[r23] Gray E. S. Madiga M. C. Hermanus T. Moore P. L. Wibmer C. K. Tumba N. L. Werner L. Mlisana K. Sibeko S. **& other authors (**2011**).** The neutralization breadth of HIV-1 develops incrementally over four years and is associated with CD4^+^ T cell decline and high viral load during acute infection. J Virol 85, 4828–4840. 10.1128/JVI.00198-11 21389135 PMC3126191

[r24] Harari A. Petitpierre S. Vallelian F. Pantaleo G. **(**2004**).** Skewed representation of functionally distinct populations of virus-specific CD4 T cells in HIV-1-infected subjects with progressive disease: changes after antiretroviral therapy. Blood 103, 966–972. 10.1182/blood-2003-04-1203 12958069

[r25] Hartikka J. Sawdey M. Cornefert-Jensen F. Margalith M. Barnhart K. Nolasco M. Vahlsing H. L. Meek J. Marquet M. **& other authors (**1996**).** An improved plasmid DNA expression vector for direct injection into skeletal muscle. Hum Gene Ther 7, 1205–1217. 10.1089/hum.1996.7.10-1205 8793545

[r26] Hessell A. J. Poignard P. Hunter M. Hangartner L. Tehrani D. M. Bleeker W. K. Parren P. W. Marx P. A. Burton D. R. **(**2009**).** Effective, low-titer antibody protection against low-dose repeated mucosal SHIV challenge in macaques. Nat Med 15, 951–954. 10.1038/nm.1974 19525965 PMC4334439

[r27] Hosie M. J. Flynn J. N. **(**1996**).** Feline immunodeficiency virus vaccination: characterization of the immune correlates of protection. J Virol 70, 7561–7568. 8892875 10.1128/jvi.70.11.7561-7568.1996 PMC190824

[r28] Hosie M. J. Addie D. Belák S. Boucraut-Baralon C. Egberink H. Frymus T. Gruffydd-Jones T. Hartmann K. Lloret A. Lutz H. **(**2009**).** Feline immunodeficiency. ABCD guidelines on prevention and management. J Feline Med Surg 11, 575–584. 10.1016/j.jfms.2009.05.006 19481037 PMC7129779

[r29] Hosie M. J. Pajek D. Samman A. Willett B. J. **(**2011**).** Feline immunodeficiency virus (FIV) neutralization: a review. Viruses 3, 1870–1890. 10.3390/v3101870 22069520 PMC3205386

[r30] Hoxie J. A. **(**2010**).** Toward an antibody-based HIV-1 vaccine. Annu Rev Med 61, 135–152. 10.1146/annurev.med.60.042507.164323 19824826

[r31] Huang G. Takeuchi Y. Korobeinikov A. **(**2012**).** HIV evolution and progression of the infection to AIDS. J Theor Biol 307, 149–159. 10.1016/j.jtbi.2012.05.013 22634206

[r32] Kraase M. Sloan R. Klein D. Logan N. McMonagle L. Biek R. Willett B. J. Hosie M. J. **(**2010**).** Feline immunodeficiency virus *env* gene evolution in experimentally infected cats. Vet Immunol Immunopathol 134, 96–106. 10.1016/j.vetimm.2009.10.015 19897254

[r33] Kwong P. D. Mascola J. R. Nabel G. J. **(**2012**).** The changing face of HIV vaccine research. J Int AIDS Soc 15, 17407. 10.7448/IAS.15.2.17407 22789610 PMC3499796

[r34] Lambotte O. Ferrari G. Moog C. Yates N. L. Liao H. X. Parks R. J. Hicks C. B. Owzar K. Tomaras G. D. **& other authors (**2009**).** Heterogeneous neutralizing antibody and antibody-dependent cell cytotoxicity responses in HIV-1 elite controllers. AIDS 23, 897–906. 10.1097/QAD.0b013e328329f97d 19414990 PMC3652655

[r35] Lang K. S. Hegazy A. N. Lang P. A. Eschli B. Löhning M. Hengartner H. Zinkernagel R. M. Recher M. **(**2007**).** “Negative vaccination” by specific CD4 T cell tolerisation enhances virus-specific protective antibody responses. PLoS ONE 2, e1162. 10.1371/journal.pone.0001162 18000535 PMC2048666

[r36] Leslie A. J. Pfafferott K. J. Chetty P. Draenert R. Addo M. M. Feeney M. Tang Y. Holmes E. C. Allen T. **& other authors (**2004**).** HIV evolution: CTL escape mutation and reversion after transmission. Nat Med 10, 282–289. 10.1038/nm992 14770175

[r37] Li Y. Svehla K. Louder M. K. Wycuff D. Phogat S. Tang M. Migueles S. A. Wu X. Phogat A. **& other authors (**2009**).** Analysis of neutralization specificities in polyclonal sera derived from human immunodeficiency virus type 1-infected individuals. J Virol 83, 1045–1059. 10.1128/JVI.01992-08 19004942 PMC2612402

[r38] Mahalanabis M. Jayaraman P. Miura T. Pereyra F. Chester E. M. Richardson B. Walker B. Haigwood N. L. **(**2009**).** Continuous viral escape and selection by autologous neutralizing antibodies in drug-naive human immunodeficiency virus controllers. J Virol 83, 662–672. 10.1128/JVI.01328-08 18987151 PMC2612349

[r39] Mascola J. R. Montefiori D. C. **(**2010**).** The role of antibodies in HIV vaccines. Annu Rev Immunol 28, 413–444. 10.1146/annurev-immunol-030409-101256 20192810

[r40] Mascola J. R. Lewis M. G. Stiegler G. Harris D. VanCott T. C. Hayes D. Louder M. K. Brown C. R. Sapan C. V. **& other authors (**1999**).** Protection of Macaques against pathogenic simian/human immunodeficiency virus 89.6PD by passive transfer of neutralizing antibodies. J Virol 73, 4009–4018. 10196297 10.1128/jvi.73.5.4009-4018.1999 PMC104180

[r41] Mascola J. R. Stiegler G. VanCott T. C. Katinger H. Carpenter C. B. Hanson C. E. Beary H. Hayes D. Frankel S. S. **& other authors (**2000**).** Protection of macaques against vaginal transmission of a pathogenic HIV-1/SIV chimeric virus by passive infusion of neutralizing antibodies. Nat Med 6, 207–210. 10.1038/72318 10655111

[r42] Medina-Ramírez M. Sánchez-Merino V. Sánchez-Palomino S. Merino-Mansilla A. Ferreira C. B. Pérez I. González N. Alvarez A. Alcocer-González J. M. **& other authors (**2011**).** Broadly cross-neutralizing antibodies in HIV-1 patients with undetectable viremia. J Virol 85, 5804–5813. 10.1128/JVI.02482-10 21471239 PMC3126317

[r43] Mikell I. Sather D. N. Kalams S. A. Altfeld M. Alter G. Stamatatos L. **(**2011**).** Characteristics of the earliest cross-neutralizing antibody response to HIV-1. PLoS Pathog 7, e1001251. 10.1371/journal.ppat.1001251 21249232 PMC3020924

[r44] Moog C. Fleury H. J. Pellegrin I. Kirn A. Aubertin A. M. **(**1997**).** Autologous and heterologous neutralizing antibody responses following initial seroconversion in human immunodeficiency virus type 1-infected individuals. J Virol 71, 3734–3741. 9094648 10.1128/jvi.71.5.3734-3741.1997 PMC191523

[r45] Moore P. L. Gray E. S. Choge I. A. Ranchobe N. Mlisana K. Abdool Karim S. S. Williamson C. Morris L. CAPRISA 002 Study Team **(**2008**).** The C3-V4 region is a major target of autologous neutralizing antibodies in human immunodeficiency virus type 1 subtype C infection. J Virol 82, 1860–1869. 10.1128/JVI.02187-07 18057243 PMC2258729

[r46] Moore P. L. Ranchobe N. Lambson B. E. Gray E. S. Cave E. Abrahams M. R. Bandawe G. Mlisana K. Abdool Karim S. S. **& other authors (**2009**).** Limited neutralizing antibody specificities drive neutralization escape in early HIV-1 subtype C infection. PLoS Pathog 5, e1000598. 10.1371/journal.ppat.1000598 19763271 PMC2742164

[r47] Muster T. Steindl F. Purtscher M. Trkola A. Klima A. Himmler G. Rüker F. Katinger H. **(**1993**).** A conserved neutralizing epitope on gp41 of human immunodeficiency virus type 1. J Virol 67, 6642–6647. 7692082 10.1128/jvi.67.11.6642-6647.1993 PMC238102

[r48] Myers G. Lenroot R. **(**1992**).** HIV glycosylation: what does it portend? AIDS Res Hum Retroviruses 8, 1459–1460. 1466982 10.1089/aid.1992.8.1459

[r49] Nomura T. Matano T. **(**2012**).** Association of MHC-I genotypes with disease progression in HIV/SIV infections. Front Microbiol 3, 234. 10.3389/fmicb.2012.00234 22754552 PMC3386493

[r50] Overbaugh J. Morris L. **(**2012**).** The antibody response against HIV-1. Cold Spring Harb Perspect Med 2, a007039. 10.1101/cshperspect.a007039 22315717 PMC3253031

[r51] Pereyra F. Palmer S. Miura T. Block B. L. Wiegand A. Rothchild A. C. Baker B. Rosenberg R. Cutrell E. **& other authors (**2009**).** Persistent low-level viremia in HIV-1 elite controllers and relationship to immunologic parameters. J Infect Dis 200, 984–990. 10.1086/605446 19656066 PMC3725728

[r52] Piantadosi A. Panteleeff D. Blish C. A. Baeten J. M. Jaoko W. McClelland R. S. Overbaugh J. **(**2009**).** Breadth of neutralizing antibody response to human immunodeficiency virus type 1 is affected by factors early in infection but does not influence disease progression. J Virol 83, 10269–10274. 10.1128/JVI.01149-09 19640996 PMC2748011

[r53] Pu R. Y. Coleman J. Omori M. Arai M. Hohdatsu T. Huang C. J. Tanabe T. Yamamoto J. K. **(**2001**).** Dual-subtype FIV vaccine protects cats against *in vivo* swarms of both homologous and heterologous subtype FIV isolates. AIDS 15, 1225–1237. 10.1097/00002030-200107060-00004 11426067

[r54] Pu R. Coleman J. Coisman J. Sato E. Tanabe T. Arai M. Yamamoto J. K. **(**2005**).** Dual-subtype FIV vaccine (Fel-O-Vax FIV) protection against a heterologous subtype B FIV isolate. J Feline Med Surg 7, 65–70. 10.1016/j.jfms.2004.08.005 15686976 PMC10911555

[r55] Rasmussen R. A. Hofmann-Lehmann R. Li P. L. Vlasak J. Schmitz J. E. Reimann K. A. Kuroda M. J. Letvin N. L. Montefiori D. C. **& other authors (**2002**).** Neutralizing antibodies as a potential secondary protective mechanism during chronic SHIV infection in CD8^+^ T-cell-depleted macaques. AIDS 16, 829–838. 10.1097/00002030-200204120-00002 11919484

[r56] Recher M. Lang K. S. Hunziker L. Freigang S. Eschli B. Harris N. L. Navarini A. Senn B. M. Fink K. **& other authors (**2004**).** Deliberate removal of T cell help improves virus-neutralizing antibody production. Nat Immunol 5, 934–942. 10.1038/ni1102 15300247

[r57] Richman D. D. Wrin T. Little S. J. Petropoulos C. J. **(**2003**).** Rapid evolution of the neutralizing antibody response to HIV type 1 infection. Proc Natl Acad Sci U S A 100, 4144–4149. 10.1073/pnas.0630530100 12644702 PMC153062

[r58] Rodriguez S. K. Sarr A. D. MacNeil A. Thakore-Meloni S. Gueye-Ndiaye A. Traoré I. Dia M. C. Mboup S. Kanki P. J. **(**2007**).** Comparison of heterologous neutralizing antibody responses of human immunodeficiency virus type 1 (HIV-1)- and HIV-2-infected Senegalese patients: distinct patterns of breadth and magnitude distinguish HIV-1 and HIV-2 infections. J Virol 81, 5331–5338. 10.1128/JVI.02789-06 17301136 PMC1900200

[r59] Rong R. Li B. Lynch R. M. Haaland R. E. Murphy M. K. Mulenga J. Allen S. A. Pinter A. Shaw G. M. **& other authors (**2009**).** Escape from autologous neutralizing antibodies in acute/early subtype C HIV-1 infection requires multiple pathways. PLoS Pathog 5, e1000594. 10.1371/journal.ppat.1000594 19763269 PMC2741593

[r60] Samman A. Logan N. McMonagle E. L. Ishida T. Mochizuki M. Willett B. J. Hosie M. J. **(**2010**).** Neutralization of feline immunodeficiency virus by antibodies targeting the V5 loop of Env. J Gen Virol 91, 242–249. 10.1099/vir.0.015404-0 19776242

[r61] Sather D. N. Armann J. Ching L. K. Mavrantoni A. Sellhorn G. Caldwell Z. Yu X. Wood B. Self S. **& other authors (**2009**).** Factors associated with the development of cross-reactive neutralizing antibodies during human immunodeficiency virus type 1 infection. J Virol 83, 757–769. 10.1128/JVI.02036-08 18987148 PMC2612355

[r62] Scheid J. F. Mouquet H. Feldhahn N. Seaman M. S. Velinzon K. Pietzsch J. Ott R. G. Anthony R. M. Zebroski H. **& other authors (**2009**).** Broad diversity of neutralizing antibodies isolated from memory B cells in HIV-infected individuals. Nature 458, 636–640. 10.1038/nature07930 19287373

[r63] Schmitz J. E. Kuroda M. J. Santra S. Simon M. A. Lifton M. A. Lin W. Khunkhun R. Piatak M. Lifson J. D. **& other authors (**2003**).** Effect of humoral immune responses on controlling viremia during primary infection of rhesus monkeys with simian immunodeficiency virus. J Virol 77, 2165–2173. 10.1128/JVI.77.3.2165-2173.2003 12525651 PMC140983

[r64] Stamatatos L. Morris L. Burton D. R. Mascola J. R. **(**2009**).** Neutralizing antibodies generated during natural HIV-1 infection: good news for an HIV-1 vaccine? Nat Med 15, 866–870. 19525964 10.1038/nm.1949

[r65] Streeck H. Brumme Z. L. Anastario M. Cohen K. W. Jolin J. S. Meier A. Brumme C. J. Rosenberg E. S. Alter G. **& other authors (**2008**).** Antigen load and viral sequence diversification determine the functional profile of HIV-1-specific CD8^+^ T cells. PLoS Med 5, e100. 10.1371/journal.pmed.0050100 18462013 PMC2365971

[r66] Tomaras G. D. Haynes B. F. **(**2009**).** HIV-1-specific antibody responses during acute and chronic HIV-1 infection. Curr Opin HIV AIDS 4, 373–379. 10.1097/COH.0b013e32832f00c0 20048700 PMC3133462

[r67] Trkola A. Purtscher M. Muster T. Ballaun C. Buchacher A. Sullivan N. Srinivasan K. Sodroski J. Moore J. P. Katinger H. **(**1996**).** Human monoclonal antibody 2G12 defines a distinctive neutralization epitope on the gp120 glycoprotein of human immunodeficiency virus type 1. J Virol 70, 1100–1108. 8551569 10.1128/jvi.70.2.1100-1108.1996 PMC189917

[r68] van Gils M. J. Euler Z. Schweighardt B. Wrin T. Schuitemaker H. **(**2009**).** Prevalence of cross-reactive HIV-1-neutralizing activity in HIV-1-infected patients with rapid or slow disease progression. AIDS 23, 2405–2414. 10.1097/QAD.0b013e32833243e7 19770692

[r69] van Gils M. J. Bunnik E. M. Burger J. A. Jacob Y. Schweighardt B. Wrin T. Schuitemaker H. **(**2010**).** Rapid escape from preserved cross-reactive neutralizing humoral immunity without loss of viral fitness in HIV-1-infected progressors and long-term nonprogressors. J Virol 84, 3576–3585. 10.1128/JVI.02622-09 20071586 PMC2838121

[r70] Veazey R. S. Shattock R. J. Pope M. Kirijan J. C. Jones J. Hu Q. Ketas T. Marx P. A. Klasse P. J. **& other authors (**2003**).** Prevention of virus transmission to macaque monkeys by a vaginally applied monoclonal antibody to HIV-1 gp120. Nat Med 9, 343–346. 10.1038/nm833 12579198

[r71] Walker L. M. Huber M. Doores K. J. Falkowska E. Pejchal R. Julien J. P. Wang S. K. Ramos A. Chan-Hui P. Y. **& other authors (**2011**).** Broad neutralization coverage of HIV by multiple highly potent antibodies. Nature 477, 466–470. 10.1038/nature10373 21849977 PMC3393110

[r72] Willett B. J. McMonagle E. L. Ridha S. Hosie M. J. **(**2006**).** Differential utilization of CD134 as a functional receptor by diverse strains of feline immunodeficiency virus. J Virol 80, 3386–3394. 10.1128/JVI.80.7.3386-3394.2006 16537606 PMC1440405

[r73] Zolla-Pazner S. Zhong P. Revesz K. Volsky B. Williams C. Nyambi P. Gorny M. K. **(**2004**).** The cross-clade neutralizing activity of a human monoclonal antibody is determined by the GPGR V3 motif of HIV type 1. AIDS Res Hum Retroviruses 20, 1254–1258. 10.1089/aid.2004.20.1254 15588347

[r74] Zwick M. B. Labrijn A. F. Wang M. Spenlehauer C. Saphire E. O. Binley J. M. Moore J. P. Stiegler G. Katinger H. **& other authors (**2001**).** Broadly neutralizing antibodies targeted to the membrane-proximal external region of human immunodeficiency virus type 1 glycoprotein gp41. J Virol 75, 10892–10905. 10.1128/JVI.75.22.10892-10905.2001 11602729 PMC114669

